# The Impact of Healthcare Insurance on the Utilisation of Facility-Based Delivery for Childbirth in the Philippines

**DOI:** 10.1371/journal.pone.0167268

**Published:** 2016-12-02

**Authors:** Hebe N. Gouda, Andrew Hodge, Raoul Bermejo, Willibald Zeck, Eliana Jimenez-Soto

**Affiliations:** 1 The University of Queensland, School of Public Health, Public Health Building, Brisbane, Queensland, Australia; 2 Queensland Centre for Mental Health Research, The Park Centre for Mental Health, Wacol, Queensland, Australia; 3 UNICEF Philippines Country Office, Manila, Philippines; 4 Institute of Tropical Medicine, Antwerp, Belgium; 5 Medical University of Graz, Department of Obstetrics and Gynaecology, Graz, Austria; 6 Abt Associates, Brisbane, Queensland, Australia; Örebro University, SWEDEN

## Abstract

**Objectives:**

In recent years, the government of the Philippines embarked upon an ambitious Universal Health Care program, underpinned by the rapid scale-up of subsidized insurance coverage for poor and vulnerable populations. With a view of reducing the stubbornly high maternal mortality rates in the country, the program has a strong focus on maternal health services and is supported by a national policy of universal facility-based delivery (FBD). In this study, we examine the impact that recent reforms expanding health insurance coverage have had on FBD.

**Results:**

Data from the most recent Philippines 2013 Demographic Health Survey was employed. This study applies quasi-experimental methods using propensity scores along with alternative matching techniques and weighted regression to control for self-selection and investigate the impact of health insurance on the utilization of FBD.

**Findings:**

Our findings reveal that the likelihood of FBD for women who are insured is between 5 to 10 percent higher than for those without insurance. The impact of health insurance is more pronounced amongst rural and poor women for whom insurance leads to a 9 to 11 per cent higher likelihood of FBD.

**Conclusions:**

We conclude that increasing health insurance coverage is likely to be an effective approach to increase women’s access to FBD. Our findings suggest that when such coverage is subsidized, as it is the case in the Philippines, women from poor and rural populations are likely to benefit the most.

## Introduction

In 2000, the Millennium Development Goals set a global target to reduce the maternal mortality ratio (MMR) by three quarters by 2015 (MDG 5), but as of 2013 a reduction of only about 42% had been reportedly achieved. Maternal mortality therefore remains a global priority. The post-2015 Sustainable Development Goals (SDGs) have proposed a new target to reduce global MMR to 70 per 100,000 livebirths by 2030 [1, 2]. The Philippines is one low and middle income country where progress has not been achieved thus far. According to UN estimates, the Philippines has a MMR of 120 per 100,000 livebirths showing no progress since 1990 when the MMR estimate was 110 per 100,000 livebirths [1].

Over 80% of maternal deaths occur as a result of events that can be prevented or treated at suitably equipped healthcare facilities and/or with the assistance of appropriately qualified professionals. Increasing the number of deliveries that take place at health centres is a crucial aspect of reducing maternal deaths [3] and the cost of delivery services is a key determinant of whether women give birth at a facility or not [4]. In recent years many developing countries have been working to improve access to delivery services through some form of insurance programme.

In 2008, the national government of the Philippines embarked upon a comprehensive strategy to reduce maternal and neonatal mortality, which included the rapid scale-up of facility-based delivery (FBD). A FBD is a delivery that takes place in a private, public or non-governmental health facility. Partly as a result of this policy, the number of institutional deliveries has risen significantly from 23% in 2003 to 61% in 2013 [5]. This rise is not consistent across all Filipino women however. The most recent Demographic Health Survey (DHS) has revealed a number of disparities with only 10% of mothers without education using facilities as compared to 84.3% of those with college education. FBD is also higher in urban vs. rural areas (72% vs. 51%) and in the richest quintile, where 91% of women delivered at a facility compared to 32.8% in the poorest quintile. Indeed other studies have reported FBD as low as 13% amongst the poorest quintile [6].

Inequities in institutional delivery are partly due to financial constraints, which have previously been identified as a critical barrier to access health care in the Philippines [7]. For many years the government has worked towards removing these barriers through social insurance. The National Health Insurance Program, was first established in 1969 and is currently administered by PhilHealth. As early as 1995, social insurance coverage was expanded to include the informal sector and the poor, but the ambitious goal of universal coverage remained elusive. In recent years, the government has adopted Universal Health Care as one of the key policy priorities. The programme has a strong focus on maternal and child health and is underpinned by an ambitious scheme to extend subsidized insurance coverage to poor families [8]. It is estimated that by 2014 PhilHealth extended subsidized coverage to 14.7 million poor families [8]. Under PhilHealth, women can access delivery services for free or at minimal cost [9, 10].

Today, more than 90% of the population is covered by PhilHealth [11]. However, coverage also remains unequally distributed across the country. Insurance coverage for women is slightly higher in rural (64%) relative to urban settings (62%) [5]. Higher coverage rates are also reported by women in the highest quintile (76%) as compared to those in the other quintiles (55% to 63%).

A systematic review of quantitative studies investigating the impact of health insurance on the use of maternal health services typically demonstrate a positive relationship [12]. However, a small number of studies, including two conducted in the Philippines, have failed to show convincing evidence of a relationship. An earlier study of DHS data found that PhilHealth coverage increased the odds of receiving at least four prenatal visits, but was not significantly associated with the odds of institutional delivery [13]. More recently, a study of the 2008 DHS in various countries, including the Philippines, found that insurance coverage was significantly associated with FBD, although household wealth, education, parity and urban residence all demonstrated a stronger influence [14]. At the same time, a recent review of qualitative studies in low and middle income countries found that while health insurance may improve women’s rate of FBD, insurance coverage does not necessarily alleviate the negative impact of physical barriers where they exist [15]. These authors also warn there are many factors influencing where a women gives birth and highlight the impact of cultural perceptions, familial influences and the perceived quality of care available. Indeed, mistreatment of women during childbirth has been identified as a significant global problem and a potential barrier to FBD [16].

The objective of this study is to examine the association between having health care insurance and women’s utilisation of facility based delivery during childbirth in the Philippines. Health system decisions, like those that influence health care insurance coverage, require reliable information on the impact policies have had on populations of interest. In order to be certain that a policy has brought about the desired change, ideally an experimental study would be conducted that allowed the comparison between those randomly exposed to the policy or intervention to a control group. Such studies are expensive and difficult to implement however. In the absence of a randomly controlled trial therefore, statistical quasi-experimental methods have been developed to make the best use of cross-sectional data like the DHS. Propensity score matching methods are statistical techniques which takes into account the covariates available in the data that are associated with receiving a so-called ‘treatment’ (in this case healthcare insurance) and by doing so reduces the confounding due to self-selection bias and allows us to be more confident of the results. There is a growing interest in propensity score matching methods [17] and are increasingly applied to the evaluation of health insurance impact on healthcare utilization [18–20]. Here we apply a quasi-experimental approach to the most recent round of DHS data (2013) to elucidate whether the average treatment effect (facility based deliveries) on the treated (the insured). To ensure results are robust to the matching process, we employ a suite of alternative methods [21].

## Methods

### Data Description

The cross-sectional survey was conducted by the Philippine National Statistics Office (NSO) from August 12, 2013 to September 24, 2013. A stratified two-stage cluster sampling scheme was employed. The survey provided representative population descriptions at the national and provincial levels as well as for urban and rural areas. Women aged 15–49 years were sampled, with a barangay (i.e. the smallest administrative unit in the country equivalent to a village or ward) or part of a barangay selected as primary sampling units. A total of 14,804 households were surveyed, with a response rate of 99.4%. The number of women surveyed totalled 16,155. The majority of questions related to pre-natal and post-natal health care and services were limited to the mother’s last birth to have occurred in the five years preceding the survey. This yields a total sample of 7,216 birth-observations. As detailed below, the nature of the question pertaining to health insurance further reduced the sample to 1,416 birth-observations. Full details on the survey are available elsewhere [5]. The publicly available dataset was obtained through online resources. The data was anonymous, with no identifiable information on the survey participants. As such internal review approval was not required.

### Variable description

In this study, the key parameter of interest is the health insurance status of the mother. Ideally, data on insurance status at the time of each child’s birth would be utilised. The survey asks respondents to detail the coverage of health insurance of each member of the household at the time of the survey. We restrict the sample to those births that have occurred in the 12 months prior to the survey date. Taking into consideration that PhilHealth enrolment in 2012 and 2013 was stable at about 78% [11], this allows us to make the assumption that current insurance status reflects that of the time of each child’s birth.

The outcome of interest is facility-based delivery. It was measured drawing on the survey question: “Where did you give birth to [name of child]?” Mothers are given the option of selecting from a list of places under the headings of home, public sector, private sector or other. We define a binary variable whereby a birth is categorised as facility-based if it was known to have occurred at a private, public or non-governmental clinic. The focus on FBD was motivated by several factors. First, all women having a delivery are in need of the service. This allows us to easily identify the sample (i.e. those in need of health services) without relying on subjective assessments of need. Secondly, FBD is an intervention that represents both the ability of the health system to supply a skilled service and the women’s ability to utilise such services under the difficult circumstance of childbirth.

The analysis relies on a set of observable socioeconomic and demographic variables derived primarily from the results of previous studies in the literature as well as data availability [4, 14, 22–25]. To capture socioeconomic status several variables were utilised. The effect of household wealth is captured by the survey supplied asset-based index [26]. The index was constructed using principal component analysis and we categorised wealth into low, middle and high tertiles. Awareness and knowledge of the benefits associated with facility-based delivery could not be directly assessed, instead these factors were gauged by the mother’s education. At least weekly viewing of television potentially measures access to information about safe delivery and has previously been shown to be associated with decreased rates of home births [27]. Formal employment might increase the range and access of information available to mothers. On the other hand, if employment is poverty-induced and indicates resource constraints it will reduce the probability of a facility-based delivery. Hence, both the mother’s and her partner’s employment are included [28].

Due to data constraints, geographic accessibility of health services was captured through a subjective indicator. The distance to a health facility was measured using a binary variable taking a value of 1 if the respondent indicated that when sick and wanting to get medical advice or treatment the distance to the health facility is a big problem and 0 otherwise. Clearly, this variable is limited. This variable does not necessarily represent the difficulty in reaching either a facility that can do a delivery or a facility known to offer quality services. Nonetheless, it still provides some indication of the barriers to health service utilization faced by women for any illness. The birth order of the child is included to proxy for maternal experience as well as the fact that first births are known to be more difficult [4].

Sociocultural factors were represented by marital status and religion. Each may influence the choice of delivery place via the influence of female autonomy, possible discrimination, and social norms, beliefs and values. We also controlled for urban-rural differences and region level heterogeneity via regional dummies. Full data description of the variables used can be found in Table 1.

**Table 1:** Data description, The Philippines Demographic and Health Survey.

Variable	Definition
Facility-based delivery	(0/1) if the birth took place in a health facility (private, public or nongovernmental)
Locality	(0/1) if household resides in a rural or urban area
Wealth	Wealth tertiles derived using principal components analysis and household assets
Mother watches TV	(0/1) if the mother watches television at least once a week
Mother’s marital status	Mother marital status: (1) Married; (2) Living together; (3) Other
Mother's education:	Mother's education attainment: (1) None; (2) Incomplete Primary; (3) Complete Primary; (4) Secondary or more
Religion	Mother's religion: (1) Catholic; (2) Protestant; (3) Islam; (4) Other
Mother’s employment	Mother’s employment status: (1) Agriculture; (2) None; (3) Manual; (4) Professional/Services
Partner’s employment	Partner’s employment status: (1) Agriculture; (2) None; (3) Manual; (4) Professional/Services
Distance to health facility	(0/1) if the mother responded to the question “Many different factors can prevent women from getting medical advice or treatment for themselves. When you are sick and want to get medical advice or treatment, is the distance to the health facility” a big problem
Child’s birth order	Birth rank of the child: (1) first-born child of the woman; (2) 2^nd^ to 4^th^ child; (3) child of birth order 5 or more
Mother's age at birth	Mother's age at the birth of the child: (1) >20; (2) 20–29; (3) 30–39; (4) 40–49
Geography	Household's regional location: (1) NCR; (2) CAR; (3) Ilocos; (4) Cagayan Valley; (5) Central Luzon; (6) CALABARZON; (7) MIMAROPA; (8) Bicol; (9) Western Visayas; (10) Central Visayas; (11) Eastern Visayas; (12) Zamboanga Peninsula; (13) Northern Mindanao; (14) Davao Peninsula; (15) SOCCSKSARGEN; (16) Caraga; (17) ARMM

### Estimation Strategy

Our empirical analysis aims to assess the impact of health insurance on the utilization of institutional delivery. Given that the uptake of insurance is unlikely to be random, we seek to approximate causal inference through a quasi-experimental approach. We regard those with health insurance as the treatment group and utilise observable characteristics and a large pool of observations to produce control observations that closely resemble the treated units [29]. Conditional on these observed characteristics, captured by the propensity scores, the selection into the treated group can be considered a random event. We employ the propensity scores along with various matching techniques as well as regression weighted methods to test the robustness of our results to alternative estimation techniques. Under the matching techniques, a comparison of the treated group with matched control group based on the propensity scores estimated will produce, on average, the estimate of the effect of insurance coverage. Under the regression method, weighting on the propensity scores as described below, is used to balance the sample on the observed characteristics. The regression, therefore, is not an end in itself but a step in the process to minimise bias. Using the propensity scores to match treated and control or to weight the regression sample makes the treated and control groups similar on all the covariates. This reduces selection bias and improves the causal interpretation of the estimated coefficient on health insurance. In both cases, our aim is to estimate the average treatment effects of health insurance on the treated (ATT).

Following Rosenbaum and Rubin [30], let *Y*_*iT*_ be the place of delivery for those *i*^th^ mothers with health insurance (‘treatment’ group), and *Y*_*iC*_ denote the place of delivery for mothers without health insurance (‘control’ group). The observed outcome can be written as $Y_{i}=(1-T_{i})Y_{i}^{C}+T_{i}Y_{i}^{T}$ where *T*_*i*_ = 0, 1 denotes treatment assignment. The gain from treatment is $\left(Y_{i}^{T}-Y_{i}^{C}\right)$ and our interest is the average effect of treatment on the treated (ATT), $E(Y_{i}^{T}-Y_{i}^{C}|T_{i}=1)$. This cannot be estimated directly since neither are normally observed as $Y_{i}^{T}$ for *T*_*i*_ = 0 and $Y_{i}^{C}$ for *T*_*i*_ = 1 are not known.

To address this problem and construct an appropriate counterfactual, we assume conditional on the observed characteristics **X**_*i*_ the decision to obtain insurance is independent of the place of delivery. This conditional independence assumption can be written as:

$$(Y_{iT},Y_{iC})⊥T_{i}|X_{i}$$ (1)

Under matching, we use propensity scores, *P*(**X**_*i*_) = Pr(*T*_*i*_ = 1|**X**_*i*_), to match treatment units with observationally similar control units. Consistent with the large literature on propensity score matching, these propensity scores are estimated using a probit model [31]. In practice, the logistic and normal distributions give similar results for partial effects and predicted probabilities (See S1 Table). Rosenbaum and Rubin [30] show that the conditional independence assumption implies (*Y*_*iT*_,*Y*_*iC*_) ⊥ *T*_*i*_|*P*(**X**_*i*_). Balancing on the propensity scores will remove selection bias due to the observables **X**_*i*_. Assuming also that the probability of obtaining insurance for the treated and control groups lay in the same domain (i.e. the region of common support), the ATT is computed as follows [32, 33]:

$$\begin{array}{l} ATET=E_{P(X_{i})}\left\{E\left(Y_{iT}|T_{i}=1,P(X_{i})\right)-E\left(Y_{iC}|T_{i}=0,P(X_{i})\right)|T_{i}=1\right\}\\ \;=\frac{1}{N^{T}}\sum_{i=1}^{N^{T}}\left[Y_{iT}^{}-\sum_{j=1}^{N^{C}}W_{ij}Y_{iC}^{}\right] \end{array}$$ (2)

where *N*^*T*^ is the number of treated units, *N*^*C*^ is the number of control units and *W*_*ij*_ is the weight given to control unit *j* in making a comparison with treated unit *i*.

A number of alternative matching estimators have been proposed in the literature [32, 34]. Three matching methods are considered. First, we employ the simple propensity-score matching estimator, where matches are made on the estimated conditional probability that observations are part of the treatment group given the covariates **X**. The standard errors are adjusted to account for the estimated treatment model parameters [35] and a caliper limit is set at 0.1. Second, we utilised the nearest neighbour matching estimator. Matches are made with control cases that most closely resemble the treatment case with regard to the set of covariates **X** using a weighted function and the Mahalanobis distance [36, 37]. At least three matches per observation are set and the matching is augmented with a regression-based adjustment to migrate any large-sample bias [36]. Standard errors derived by Abadie and Imbens [35], Abadie and Imbens [36], Abadie and Imbens [37] are applied as it is known that conventional bootstrap methods do not yield valid estimates. Finally, we estimated the nonparametric kernel matching estimator [32]. Matches are assigned according to a kernel function of the predicted propensity scores. A normal density function is set and a caliper limit is set at 0.1.

As noted above, an alternative technique is to use standard probability models to estimate the effect of insurance on institutional delivery, while using the propensity scores to weight the sample [38–40]. We follow the weighted-regression method proposed by Hirano, Imbens [41], which we adapt to the simple probit case. We implement the approach by considering an underlying continuous but latent response variable $y_{i}^{*}$ and the regression:

$$y_{i}^{*}=\beta_{0}+\beta_{1}T_{i}+\beta X_{i}+\varepsilon_{it}\;(i=1,\ldots ,N)$$ (3)

The observed variable *y*_*i*_, is linked to *y*^*∗*^ via the response mechanism:

$$y_{i}=\{\begin{array}{c} 1\;if\;y^{*}>0\\ 0\;if\;y^{*}\leq 0 \end{array}$$ (4)

Assuming that *ε* ∼ *N*(0, 1), the probability of a facility-based delivery is given by:

$$\mathrm{Pr}\left(y_{i}=1|T_{i},X\right)=\Phi \left(\beta_{0}+\beta_{1}T_{i}+\beta X_{i}\right)$$ (5)

where Φ(.) is the cumulative standard normal distribution function. (While we have assumed that the variance of *ε* is 1 (probit model), it is common also to assume that variance is *π*^2^*/*3 (logit model)). The weighted probit model is estimated using unity weights for treated units and $\hat{P}(X)/[1-\hat{P}(X)]$ for control units [42], where $\hat{P}(X)$ is a consistent estimate of *P*(**X**_*i*_) = Pr(*T*_*i*_ = 1|**X**_*i*_) and $0<\hat{P}(X)<1$ (One could use the weights $1/\hat{P}(X)$ for the treated and $1/[1-\hat{P}(X)]$ for the control group if one is interested in computing an estimate mirroring the average treatment effect). The average partial effect (i.e. discrete difference) on *T*_*i*_ is computed to gauge the impact of health insurance on facility-based deliveries. The standard errors are adjusted for clustering at the survey’s primary sample unit to take into account the survey sample design and correlation that exists among households who live in the same barangay or part of a barangay.

The reliability of the estimations for both the matching and weighted regression techniques relies on the balancing of the covariates between the treatment and control groups. Following Rosenbaum and Rubin [43], we employed two balancing tests. First, we computed the standardized bias before and after matching. The bias is computed as the percentage difference of the sample means in the treated and matched control groups as a percentage of the square root of the average of the sample variances in both groups. While no consensus exists on a level of standardized difference that would indicate imbalance, a difference of less than 10 percent is taken to indicate negligible differences [23, 44]. Second, we computed the pseudo *R*^*2*^ and likelihood-ratio test of joint insignificance of all regressors from a probit estimation of the conditional treatment probability before and after matching [45]. There should be no systematic difference in the distribution of the regressors after matching. Thus, the *LR* test should not reject the null hypothesis of joint insignificance and the pseudo *R*^*2*^ should be fairly low. Finally, we estimated the ATT for different subsamples to test for heterogeneous impacts and the robustness of the base results.

#### Ethical Issues

The datasets used in this study were obtained from the MEASURE DHS website http://dhsprogram.com. Full review of this study from an institutional review board was not sought as this manuscript involved secondary data analysis of datasets that are publicly available, with no identifiable information on the survey participants.

## Results

### Characteristics of the Sample

Table 2 presents the characteristics of insured and uninsured households that have experienced a birth of a child in the 12 months prior to the survey date. The table provides numbers and proportions for both matched and unmatched data demonstrating that 96.2% of insured and 99.7% of uninsured are successfully matched.

**Table 2:** Summary statistics on covariates by insurance coverage before and after matching, The Philippines.

Variable	Unmatched				Matched			
	Insured		Uninsured		Insured		Uninsured	
	N	%	N	%	N	%	N	%
Locality								
Rural	460	0.59	374	0.59	445	0.59	371	0.59
Urban	322	0.41	260	0.41	309	0.41	257	0.41
Mother watches television	592	0.76	434	0.69	565	0.75	482	0.76
Wealth								
Low	328	0.42	329	0.52	323	0.43	263	0.42
Middle	228	0.29	217	0.34	224	0.30	177	0.28
High	226	0.29	88	0.14	205	0.27	192	0.30
Mother's Marital Status								
Married	576	0.74	276	0.44	546	0.73	457	0.72
Living together	154	0.20	302	0.48	154	0.21	129	0.20
Other	52	0.07	56	0.09	52	0.07	46	0.07
Mother's Education								
None	7	0.01	11	0.02	7	0.01	7	0.01
Incomplete Primary	74	0.10	85	0.13	74	0.10	56	0.09
Complete Primary	68	0.09	66	0.10	67	0.09	58	0.09
Secondary or more	633	0.81	472	0.74	604	0.80	511	0.81
Religion								
Catholic	573	0.73	448	0.71	549	0.73	451	0.71
Protestant	52	0.07	38	0.06	51	0.07	36	0.06
Islam	74	0.10	84	0.13	72	0.10	68	0.11
Other	83	0.11	64	0.10	80	0.11	78	0.12
Mother's Employment								
Agriculture	422	0.54	429	0.68	416	0.55	346	0.55
None	63	0.08	37	0.06	62	0.08	46	0.07
Manual	50	0.06	45	0.07	50	0.07	37	0.06
Professional	247	0.32	123	0.19	224	0.30	203	0.32
Partner's Employment								
Agriculture	10	0.01	11	0.02	10	0.01	10	0.02
None	227	0.29	216	0.34	223	0.30	173	0.27
Manual	279	0.36	265	0.42	274	0.36	234	0.37
Professional	228	0.29	115	0.18	207	0.28	181	0.29
Partner's missing information	38	0.05	27	0.04	38	0.05	33	0.05
Distance to health facility	266	0.34	240	0.38	261	0.35	219	0.35
Child's birth order								
1	215	0.28	237	0.37	211	0.28	176	0.28
2–4	394	0.50	315	0.50	380	0.51	324	0.51
>4	173	0.22	82	0.13	161	0.21	132	0.21
Mother's age at birth								
<20	64	0.08	116	0.18	64	0.09	63	0.10
20–29	367	0.47	367	0.58	364	0.48	283	0.45
30–39	299	0.38	135	0.21	281	0.37	238	0.38
40–49	52	0.07	16	0.03	43	0.06	49	0.08
Geography								
NCR	77	0.10	74	0.12	76	0.10	56	0.09
CAR	37	0.05	25	0.04	37	0.05	30	0.05
Ilocos	31	0.04	25	0.04	30	0.04	24	0.04
Cagayan Valley	38	0.05	25	0.04	37	0.05	31	0.05
Central Luzon	67	0.09	50	0.08	67	0.09	65	0.10
CALABARZON	62	0.08	59	0.09	61	0.08	45	0.07
MIMAROPA	27	0.04	30	0.05	27	0.04	28	0.05
Bicol	46	0.06	40	0.06	45	0.06	30	0.05
Western Visayas	54	0.07	42	0.07	54	0.07	39	0.06
Central Visayas	36	0.05	42	0.07	36	0.05	33	0.05
Eastern Visayas	33	0.04	17	0.03	28	0.04	33	0.05
Zamboanga Peninsula	39	0.05	32	0.05	38	0.05	37	0.06
Northern Mindanao	45	0.06	17	0.03	37	0.05	39	0.06
Davao Peninsula	52	0.07	27	0.04	44	0.06	38	0.06
SOCCSKSARGEN	40	0.05	31	0.05	39	0.05	28	0.04
Caraga	55	0.07	34	0.05	53	0.07	39	0.06
ARMM	43	0.06	64	0.10	43	0.06	36	0.06
Observations	782		634		752		632	
Pseudo *R*^*2*^, Raw Sample	0.16							
Pseudo *R*^*2*^, Matched Sample	0.01							
*LR* Stat., Raw Sample	309.77	[0.000]						
*LR* Stat., Matched Sample	20.61	[0.997]						
Mean bias, Raw Sample	13.1							
Mean bias, Matched Sample	3							

Using the propensity scores to account for the dissimilarities between the insured and uninsured births, the average differences in the characteristics between the groups decreases from a mean of 13.1% to 3%. Furthermore, the probit regression on the propensity score weighted sample is estimated with a relatively low pseudo *R*^*2*^ of 0.01 and the likelihood ratio statistic does not reject the null hypothesis of joint insignificance of all regressors on the conditional treatment probability. Such results suggest that covariate balance is satisfied.

Those women who were not covered by health insurance were more likely to be unmarried, have less than complete primary education, work in agriculture and be younger, with fewer children, than those with insurance. Those missing information on their partner’s employment status made up almost 5% of the sample.

### Insurance and FBD

Table 3 presents the proportion of women who delivered in a facility by insurance coverage before and after matching. Overall, the proportion of women in this sample who delivered their child in a facility is low at 64.3%. The differences in the proportions of facility-based deliveries between the uninsured and insured hold with and without accounting for propensity score weights. In general, the proportion of births that took place in a facility is higher for women who were insured (70.3%—SD 0.46) versus uninsured (56.9%—SD 0.5). This observation remains valid for rural and urban as well poor and non-poor subpopulations. Initial differences in the group-specific means can be observed across various dimensions of the unmatched data. Poor and uninsured (43%—SD 0.5) are least likely to have a facility based delivery followed by the rural uninsured (50%—SD 0.5) while the urban and non-poor are the most likely (78%—SD 0.41 and 84%—SD 0.37 respectively).

**Table 3:** Summary statistics on the facility-based deliveries by insurance coverage before and after matching, The Philippines.

	Unmatched									Matched								
	Insured			Uninsured			All			Insured			Uninsured			All		
	N	Mean	SD	N	Mean	SD	N	Mean	SD	N	Mean	SD	N	Mean	SD	N	Mean	SD
*Locality*	* *									* *								
Rural	460	0.65	0.48	374	0.5	0.5	834	0.58	0.49	445	0.65	0.48	371	0.5	0.5	816	0.58	0.49
Urban	322	0.78	0.41	260	0.67	0.47	582	0.73	0.44	303	0.78	0.42	257	0.68	0.47	560	0.73	0.44
																		
*Wealth*																		
Poor	328	0.52	0.5	329	0.43	0.5	657	0.48	0.5	304	0.53	0.5	319	0.43	0.5	623	0.48	0.5
Non-poor	454	0.84	0.37	304	0.72	0.45	758	0.79	0.41	440	0.84	0.37	289	0.72	0.45	729	0.79	0.41
																		
**Total**	**782**	**0.7**	**0.46**	**634**	**0.57**	**0.5**	**1416**	**0.64**	**0.48**	**752**	**0.7**	0.46	632	**0.57**	0.5	1384	**0.64**	0.48

N = Number of births; Mean = mean number of facility births for specified sample; SD = Standard Deviation from the mean

### Estimating the impact of Insurance on FBD

The first step of propensity score matching is to address the potential association between the variables available and the likelihood of having insurance. To do this both logistic and probit regressions were conducted to ensure that similar results were obtained (see S1 Table). The regression analysis provides us with the covariates that are likely to contribute to selection bias. The results indicate that insured households are generally wealthier, with members employed in professional occupations. In addition, mothers from insured households are more often married, with higher levels of educational attainment, more children and older at the time of birth compared to uninsured households. A significant association was also observed between those birth events where the partner’s information was missing and insurance coverage. This is probably because those missing partner’s information were more likely to be in the top three wealth quintiles than those with data (68% versus 46% respectively). To address the potential bias arising from this missing data it was therefore included as a covariate.

Using the propensity scores to address potential selection bias of households covered by insurance allowed us to estimate the impact of insurance on facility based deliveries or the average treatment effect on the treated (ATT). As reported in Table 4, the ATT have the expected signs and the results are robust to the alternative estimation techniques used; propensity score, nearest neighbour and kernel matching. Apart from one specification, nearest neighbour matching, the ATT results are all significant to at least a 5% level. The consistent direction and relative size of the findings using different methods suggests that our results are robust and that having insurance does increase the likelihood of institutional delivery amongst this sample.

**Table 4:** Average treatment effect on the treated of insurance on the utilization of facility-based delivery using propensity score weighting or matching, The Philippines.

	Alternative Matching Methods						Propensity Score Weighted			
	Propensity Score		Nearest Neighbour		Kernel-PS matching		Ordered Probit, unadjusted		Ordered Probit, adjusted	
	ATT	SE	ATT	SE	ATT	SE	ATT	SE	ATT	SE
All	0.0973***	(0.035)	0.0547*	(0.028)	0.0786**	(0.032)	0.07**	(0.031)	0.0802***	(0.026)
*Locality*										
Rural (N = 816)	0.1127**	(0.051)	0.0873**	(0.037)	0.0964**	(0.039)	0.0905**	(0.038)	0.091***	(0.032)
Urban (N = 560)	0.0486**	(0.025)	0.0764	(0.048)	0.0648	(0.054)	0.0622	(0.051)	0.0473	(0.036)
*Wealth*										
Poor (N = 623)	0.1102**	(0.046)	0.0868*	(0.046)	0.087	(0.053)	0.0936**	(0.042)	0.1011**	(0.039)
Non-poor (N = 729)	0.053	(0.035)	0.0576	(0.037)	0.0683*	(0.04)	0.064*	(0.034)	0.0614**	(0.029)

*Notes*: Dependent variable is facility-based delivery. Standard errors are in parentheses. For parametric specifications, they are clustered at the primary sampling unit. For PS and NN matching, Abadie and Imbens (2006, 2011, 2012) derived standard errors are used. For Kernel-PS matching, bootstrapped standard errors using 1,000 replications of the sample are used.

(*), (**), and (***) denote significance at 10%, 5%, and 1% levels, respectively. ATT, average treatment effect on the treated; S.E., standard error; PS, propensity score; NN, nearest neighbour.

Table 4 also presents the ATT stratifying the sample by rural-urban and non-poor—poor sub-populations. There is notable heterogeneity in the observed impact, with all treatment effects being larger for the rural and poor sub-populations. In other words rural and poor women with insurance coverage are 9 to 11 per cent more likely to have a FBD than those uninsured. For the rural sub-sample, the ATTs are statistically significant for all specifications, while for the urban sub-population it is only significant for one specification at conventional levels. The statistical significance of the ATTs is more robust across specifications for the poor samples. In short, the effect of insurance on institutional delivery appears to be positive across the population but greater amongst poor and rural within the Philippines.

## Conclusions for Practice

Our results suggest that women who gave birth in the previous year were more likely to use FBD services if they were covered by insurance. This outcome was particularly significant when looking at women from poor or rural households. Earlier studies have argued that insurance coverage, particularly amongst the poor, was yet to translate into access to services due to various factors including the community’s lack of awareness of the rights to access health services [7, 46]. In contrast, our results suggest that insurance coverage for women in poor and rural households is possibly leading to an increasing demand for services and higher probabilities of FBD. This is an encouraging finding as it indicates that the country is making progress to address financial barriers that had previously prevented insured disadvantaged groups from effectively accessing health services [47].

Our findings point to a positive impact of insurance status on healthcare utilization. This is consistent with the literature emerging from other lower and middle-income countries. Of the fourteen analyses examining the relationship between insurance and institutional delivery considered in the 2013 review by Comfort et al, [12] only four studies indicated that insurance had no effect on institutional delivery. Amongst these was Kozhimannil’s et al. investigation which compared DHS data from 1998 and 2003; pre and post PhilHealth scale-up [13]. It revealed a lack of significant association between insurance and facility-based delivery, partly explained by logistical barriers that insurance cannot alleviate; such as the reliance on family members to care for older children while the woman is in hospital. More importantly, while insurance coverage may have increased rapidly in the period studied, 2003 may have been too soon after the scale-up to observe a change.

The dearth of experimental and longitudinal data on the impact of insurance has to some extent hampered efforts to evaluate the impact of health insurance on healthcare utilization. Of those studies relying on cross-sectional data, only a few have exploited available statistical techniques to deal with biases such as those arising from self-selection, as we have done in this paper. These studies have mostly demonstrated a positive effect of insurance on the presence of skilled birth attendants and hospital delivery [48–50].

Our study highlights the importance of providing insurance coverage to specific subsets of the population; in our case women that are poor and living in rural areas, especially in the context of the drive to provide universal health coverage (UHC). In the absence of health insurance, out-of-pocket expenditures can force households into poverty or to forego essential services and this is especially true for those who are already poor. There is now consensus that public financing is required to address inequities in health and achieve UHC by extending insurance coverage to disadvantaged populations so that they no longer face financial barriers to access essential health services [51]. This is particularly relevant to women for whom cash is typically less accessible than for men [52].

A recent analysis suggests that insurance coverage over the 2008–2013 period increased across all quintiles and the distribution became more pro-poor [8]. Our results also suggest that for poor and rural women, having access to insurance led to increased utilization of FBD. Similar findings have been reported in other countries with regards to institutional delivery [53–55] as well as other healthcare services [56–59]. In settings with persistent inequities, however, it is paramount that disadvantaged groups of the population are targeted in the scale-up of social insurance, as the Philippines has done, and that these reforms are complemented by programmes addressing other barriers to healthcare access (ie physical and cultural barriers)

Our results on the impact of insurance coverage amongst women on FBD are robust to a range of statistical methods. We have used propensity scores along with alternative matching methods and weighted regression to control for selection bias based on observables. However, the usual caveats to the use of propensity scores when examining cross-sectional datasets apply. First, the data used here is cross-sectional and therefore cannot be employed to infer causality [60]. Second, a note of caution is needed when interpreting results where variables related to the exposure are similar to those related to the outcome [61, 62]. Third, importantly, our study aims to measure the impact that insurance has on FBD coverage, but due to lack of data we cannot examine whether the observed increased in coverage has been at the expense of quality of care. Since improvements in health outcomes are dependent on the quality of care provided, this issue should be addressed by future studies. Indeed, though facility-based delivery has increased in the Philippines, maternal mortality has not seen a commensurate improvement over the same time period and this warrants further investigation. Future research will also be necessary to evaluate the longer-term affects and sustainability of the PhilHealth insurance scheme.

Despite these limitations, ours is the first study to report quasi-experimental evidence of the positive impact that insurance coverage is likely to have had on institutional delivery in Philippines, a country with stubbornly high maternal mortality rates. By international standards, the current rates of institutional delivery remain low in the Philippines. If UHC goals are to be achieved and the equity gaps in the Philippines are to be closed, findings from this analysis suggest that the current efforts to provide subsidized insurance coverage to the poor and the vulnerable population should be sustained so as to encourage facility based childbirth, a key preventative factor in reducing maternal deaths and improving child outcomes. Indeed, as a key target of the SDGs, UHC is a priority globally. Our study demonstrates how other countries can use appropriate survey data, like DHS, to effectively investigate the impact of health policies, like insurance schemes, on health care utilisation.

## Supporting Information

S1 Table Regressions of probability of the mother being covered by insurance. (DOCX)
