# Supplementary material for: The Impact of Healthcare Insurance on the Utilisation of Facility-Based Delivery for Childbirth in the Philippines
Source: PLoS One. 2016 Dec 2;11(12):e0167268. doi: 10.1371/journal.pone.0167268 (PMC5135090; doi:10.1371/journal.pone.0167268)
Supplement: S1 Table — (DOCX) [file pone.0167268.s001.docx]

**Table S1:** Regressions of probability of the mother being covered by insurance.

| **Variable** | **Probit** | | | |  | **Logit** | | |
| --- | --- | --- | --- | --- | --- | --- | --- | --- |
|  |  | **Coeff.** |  | **S.E.** |  | **Odds** |  | **S.E.** |
| Constant |  | -0.4038 |  | (0.411) |  |  |  |  |
| Urban |  | 0.0081 |  | (0.093) |  | 0.9957 |  | (0.154) |
| Mother watches television |  | 0.1683 | * | (0.093) |  | 1.3243 | * | (0.206) |
| Wealth |  |  |  |  |  |  |  |  |
| Middle |  | 0.0843 |  | (0.098) |  | 1.1694 |  | (0.192) |
| High |  | 0.4765 | *** | (0.127) |  | 2.2706 | *** | (0.486) |
| Mother's Marital Status |  |  |  |  |  |  |  |  |
| Living together |  | -0.8519 | *** | (0.089) |  | 0.2472 | *** | (0.037) |
| Other |  | -0.9898 | *** | (0.219) |  | 0.1910 | *** | (0.071) |
| Mother's Education: |  |  |  |  |  |  |  |  |
| Incomplete Primary |  | -0.0222 |  | (0.341) |  | 0.9598 |  | (0.550) |
| Complete Primary |  | 0.0694 |  | (0.348) |  | 1.1262 |  | (0.658) |
| Secondary or more |  | 0.1633 |  | (0.337) |  | 1.3046 |  | (0.740) |
| Religion |  |  |  |  |  |  |  |  |
| Protestant |  | -0.0857 |  | (0.158) |  | 0.8552 |  | (0.222) |
| Islam |  | -0.2813 |  | (0.191) |  | 0.6303 |  | (0.200) |
| Other |  | -0.0171 |  | (0.122) |  | 0.9773 |  | (0.202) |
| Mother's Employment |  |  |  |  |  |  |  |  |
| None |  | -0.1639 |  | (0.154) |  | 0.7754 |  | (0.197) |
| Manual |  | -0.2450 |  | (0.206) |  | 0.6761 |  | (0.231) |
| Professional |  | 0.0842 |  | (0.169) |  | 1.1752 |  | (0.332) |
| Partner's Employment |  |  |  |  |  |  |  |  |
| None |  | 0.0648 |  | (0.307) |  | 1.0904 |  | (0.574) |
| Manual |  | 0.0150 |  | (0.101) |  | 1.0202 |  | (0.171) |
| Professional |  | 0.2900 | ** | (0.121) |  | 1.6185 | ** | (0.327) |
| Partner's missing information |  | 0.8858 | *** | (0.287) |  | 4.4325 | *** | (2.143) |
| Distance to health facility |  | -0.0046 |  | (0.081) |  | 0.9969 |  | (0.134) |
| Child's birth order |  |  |  |  |  |  |  |  |
| 2-4 |  | 0.1809 | * | (0.097) |  | 1.3608 | * | (0.220) |
| >4 |  | 0.5408 | *** | (0.148) |  | 2.4333 | *** | (0.599) |
| Mother's age at birth |  |  |  |  |  |  |  |  |
| 20-29 |  | 0.0245 |  | (0.124) |  | 1.0536 |  | (0.221) |
| 30-39 |  | 0.2400 |  | (0.151) |  | 1.5224 | * | (0.385) |
| 40-49 |  | 0.3486 |  | (0.234) |  | 1.8781 |  | (0.754) |
| Geography |  |  |  |  |  |  |  |  |
| CAR |  | 0.1321 |  | (0.217) |  | 1.2321 |  | (0.443) |
| Ilocos |  | 0.1851 |  | (0.232) |  | 1.3273 |  | (0.519) |
| Cagayan Valley |  | 0.2953 |  | (0.226) |  | 1.6279 |  | (0.615) |
| Central Luzon |  | 0.1147 |  | (0.174) |  | 1.1985 |  | (0.351) |
| CALABARZON |  | 0.2357 |  | (0.171) |  | 1.4801 |  | (0.419) |
| MIMAROPA |  | 0.0683 |  | (0.227) |  | 1.1471 |  | (0.434) |
| Bicol |  | 0.3255 |  | (0.201) |  | 1.6774 |  | (0.563) |
| Western Visayas |  | 0.2664 |  | (0.194) |  | 1.5457 |  | (0.497) |
| Central Visayas |  | 0.0384 |  | (0.198) |  | 1.0592 |  | (0.352) |
| Eastern Visayas |  | 0.3206 |  | (0.244) |  | 1.7612 |  | (0.736) |
| Zamboanga Peninsula |  | 0.3125 |  | (0.209) |  | 1.6724 |  | (0.584) |
| Northern Mindanao |  | 0.7811 | *** | (0.222) |  | 3.7329 | *** | (1.412) |
| Davao Peninsula |  | 0.5961 | *** | (0.204) |  | 2.6821 | *** | (0.916) |
| SOCCSKSARGEN |  | 0.3467 |  | (0.212) |  | 1.7855 | * | (0.623) |
| Caraga |  | 0.5450 | *** | (0.203) |  | 2.4802 | *** | (0.834) |
| ARMM |  | 0.0312 |  | (0.261) |  | 1.0668 |  | (0.457) |
| Observations |  | 1,416 |  |  |  | 1,416 |  |  |
| Log likelihood |  | 309.770 |  |  |  | 310.71 |  |  |
| Pseudo *R^2^* |  | 0.1591 |  |  |  | 0.1595 |  |  |
| *Notes:* Dependent variable is insurance status of the mother and the unit of observation child delivery. Coeff., coefficient; S.E., standard error. | | | | | | | | |
